# What Factors Predict Adaptive Functioning in Preschool Children with Autism Spectrum Disorder? A Longitudinal Study

**DOI:** 10.3390/jcm13061565

**Published:** 2024-03-09

**Authors:** Laura Casula, Maria Grazia Logrieco, Giulio D’Urso, Silvia Guerrera, Emanuela Petrolo, Ilaria Nicolì, Vittoria Celentano, Giusi Antonia Toto, Stefano Vicari, Mirco Fasolo, Giovanni Valeri

**Affiliations:** 1 Department of Neuroscience, IRCCS Children’s Hospital Bambino Gesù, Piazza Sant’Onofrio, 4, 00165 Rome, Italy; 2 Department of Human Sciences, University of Foggia, Via Arpi 55, 71121 Foggia, Italy; 3 Department of Neuroscience, Imaging and Clinical Sciences, University “G. d’Annunzio” Chieti-Pescara, Via dei Vestini 33, 66100 Chieti, Italy

**Keywords:** adaptive functioning, preschoolers, autism, repetitive and restricted behaviors, longitudinal study

## Abstract

Adaptive functioning constitutes a fundamental aspect of the phenotype associated with autism spectrum disorder (ASD) in preschool-aged children, exerting a significant influence on both the child and the family’s overall quality of life. The aim of this study was to investigate the predictors of the adaptive functioning domains in preschool-age children with ASD at two time points, providing a snapshot of this fundamental developmental step. **Methods:** Ninety-five children with ASD (*M* = 3.89, *SD* = 1.13) were included in the study and longitudinal data (the mean length of the longitudinal data collection was 1 year) on ASD features such as social communication and social interaction, repetitive and restricted behavior, cognitive level, and adaptive functioning were collected. We considered autistic features, cognitive level, and sociodemographic factors as possible predictors of the different adaptive functioning domains one year later. **Results:** Data obtained showed a worsening of the ASD features and adaptive functioning after one year. Furthermore, the severity of repetitive and restricted behavior predicted adaptive functioning, especially in the social and practical domains of the child, one year later. This prediction was observed alongside the child’s cognitive level. **Conclusions:** The study identifies some potential predictive factors of specific adaptive functioning domains in preschoolers with ASD. Considering how critical adaptive functioning is for the well-being of both the child and their family, it becomes imperative to design early-stage interventions focused on nurturing adaptive skills in children with ASD.

## 1. Introduction

Autism spectrum disorder (ASD) is a neurodevelopmental disorder characterized by socio-communicative abnormalities and restricted, repetitive, and stereotyped patterns of behavior and interest (RRBs) [1]. Together with the core symptoms, co-occurring psychiatric or neurological disorders and intellectual disabilities are common in children with autism. These two sets of symptoms have a wide range of severity levels, which may be different for each child with ASD [2]. Hence, it is important to note that there is a wide range of heterogeneity within the autism spectrum, and individuals with ASD may have varying levels of adaptive functioning abilities [2,3]. Adaptive functioning refers to an individual’s ability to manage and adapt to the demands of everyday life, and includes skills such as socialization, communication, self-care, home living, and leisure and community participation. Individuals with ASD may encounter challenges in these domains, and studies have indicated that a significant number of such individuals demonstrate limitations in their adaptive functioning [4,5,6]. Deficit in adaptive functioning is more prevalent in preschool-aged and school-aged children with ASD than in non-ASD children, and also more prevalent in children with other neurodevelopmental conditions [7,8,9]. The purpose of this study is to explore and identify factors that predict adaptive functioning domains in preschool-aged children with ASD at two different time points. The aim is to capture a dynamic picture of the developmental trajectory in preschool-aged children with ASD. This allows for a more comprehensive understanding of the factors that influence adaptive functioning in these children over time. We investigated some potential components, such as cognitive abilities, ASD core symptoms, children’s age, and socioeconomic factors. Moreover, we analyzed different domains of adaptive functioning, including, conceptual, practical, and social adaptive functioning. The study aims to provide valuable insights into the complex interplay of factors that contribute to the adaptive functioning of preschool-aged children with ASD. Understanding these predictors can inform the development of targeted interventions and support strategies to enhance adaptive skills in this population, ultimately improving their overall quality of life and developmental outcomes.

### 1.1. Predictors of Adaptive Functioning

Different studies conducted on children showed that some clinical features, such as cognitive skills, severity of autism core symptoms, age of the child, and familiar socioeconomic status (SES), could be predictors of later adaptive functioning [9,10]. In this context, longitudinal studies are important for understanding the developmental trajectory of the disorder and its associated challenges in adaptive functioning. However, results are still limited and have yielded mixed results [5,11,12]. Cognitive skills are among the most studied predictors of adaptive functioning. Difficulties in adaptive functioning are a fundamental requirement for diagnosing intellectual disability (ID), in addition to an intellectual impairment (e.g., IQ < 70). Typically, the presence of ID alongside ASD indicates a less favorable prognosis [13], and a childhood IQ < 70 tends to result in poorer outcomes in adulthood [14]. More specifically, within individuals diagnosed with both ASD and ID, lower IQ is linked to inferior adaptive functioning. However, research suggests that the opposite is true; individuals with ASD who are cognitively capable still exhibit adaptive skills significantly below what would be expected based on their intellectual capacity [15,16]. In fact, ASD individuals with an IQ > 70 demonstrate a significant discordance between cognitive ability and adaptive functioning [17,18]. While different studies with preschool-aged children, e.g., [5,19,20,21,22], highlighted the significant impact of cognitive skills on adaptive functioning, findings show that adaptive functioning may serve as a more reliable indicator of future ability to adapt than factors related to diagnosis and cognitive abilities [23]. Impairments in adaptive functioning are not fully explained by cognitive abilities [5,20,24,25], suggesting that other factors or clinical features may contribute to adaptive functioning outcome in children with ASD. Beyond cognitive skills, advanced early adaptive functioning and linguistic abilities of children have been found to predict their subsequent adaptive functioning outcomes and developmental pathways [26,27,28,29,30]. Nonetheless, in a recent investigation involving 2225 infants, children, and adolescents (aged 1 to 18 years), although enhanced cognitive abilities were linked to improved adaptive functioning, the age at diagnosis was found to account for a greater extent of variability in adaptive functioning compared to cognitive ability alone [17]. Other predictors of adaptive functioning to consider in children with ASD are the severity of the symptomatology and the core symptoms. Research consistently shows a positive association between social interest and adaptive functioning in children with ASD [31,32]. Decreased social interest in children with ASD can significantly impact their ability to learn from everyday experiences. For example, if a child with ASD does not observe another person performing a routine task like washing hands, it becomes more challenging for the child to imitate the action and acquire the skill. Even the existence of RRBs significantly decreases children’s involvement in various activities, particularly in the areas of community mobility and household tasks, thereby distinguishing these individuals and raising the likelihood of decreased engagement in daily routines [33,34,35,36]. In a cohort of preschool-aged children with ASD, the membership to distinct trajectories of adaptive functioning was influenced by ASD severity, cognitive functioning, language skills, age at diagnosis, and gender [28]. A study on a large cohort (i.e., 5–10 years old) found that symptom severity was poorly associated with adaptive functioning [37], while several investigations found that higher deficits in social communication interaction predicted lower levels of adaptive functioning, even in samples of individuals across a range of ages [5,6,31]. These data show that while RRBs are core features of ASD, they may not have as strong a relationship with adaptive functioning outcomes as social communication skills. On the other hand, other studies showed the predictive power of RRBs on later adaptive functioning [38] and that the impact of RRBs was stronger in childhood compared to toddlerhood [35]. An investigation conducted by Yang et al. [39] focused on 77 preschool-aged children with ASD. The study revealed that age and nonverbal ability played a significant role in accounting for variance in adaptive functioning. In contrast, ASD symptom severity did not contribute to adaptive outcomes beyond these factors. This aligns with earlier findings from a study of 125 toddlers by Ray-Subramanian et al. [40], where variability in adaptive functioning was influenced by age and cognitive ability, but not ASD symptom severity. In addition to symptom severity and cognitive ability, studies have explored the association between adaptive outcomes in ASD with socioeconomic status (SES). Parents’ education and employment can have an impact on children’s adaptive functioning. Although research on the influence of SES on adaptive outcomes in young children with ASD is still relatively new, a higher SES has been linked to earlier diagnosis [41]. This early diagnosis could potentially lead to enhanced adaptive functioning through early intervention efforts. Additionally, SES appears to be directly associated with parental well-being and competence, which in turn influence adaptive functioning [42]. Hodge et al. [22], in a cohort of 99 preschool-aged children with ASD, showed that cognitive ability and SES significantly explained variance in both domain-specific and global adaptive functioning. In contrast, ASD symptom severity did not predict variability in adaptive functioning components. Moreover, Neufeld and colleagues [36] found a significant link between RRBs and adaptive functioning, and identified familial factors such as maternal education and parental stress as strong predictors of adaptive functioning in individuals with ASD. A previous research endeavor examining the repercussions of SES on autistic symptoms revealed a correlation between lower SES and heightened emotional and behavioral difficulties, potentially exacerbating adaptive behaviors in individuals with ASD [43]. Recent studies showed an impact of maternal education on children’s adaptive skills [30] and no substantial correlation between SES and adaptive behaviors in preschool-aged children with ASD [42]. In cases of individuals with ASD, in contrast to neurotypical children [44], family socioeconomic status was found to predict adaptive outcomes in adolescence and adulthood [45]. 

### 1.2. The Current Study

Adaptive functioning in preschool aged children with ASD has been widely explored. However, longitudinal studies are scarce and show mixed results. The existing body of research indicates that there are few factors that have a strong relationship with adaptive functioning outcomes, such as cognitive and language abilities. On the other hand, other factors, such as core features of ASD and SES, may play a more significant role in determining adaptive functioning outcomes for these children. However, to date, no longitudinal study has simultaneously looked at adaptive functioning domains, ASD core symptoms, cognitive abilities, and SES as predictors of adaptive functioning domains. The present longitudinal study aims to examine the predictive factors associated with different domain-specific and global components of adaptive functioning in preschool-aged children with ASD at two time points. Specifically, we examined whether factors such as ASD core symptoms severity and adaptive abilities measured at two time points, cognitive abilities, and SES could be predictive of one-year-later adaptive functioning. The examination of the effect of development on specific adaptive skills and the identification of the associated factors in very young children with ASD is fundamental for the understanding of predictors, outcomes, and intervention. Evaluating adaptive functioning serves as a valuable addition to the diagnostic assessment for individuals believed to be dealing with ASD, offering crucial insights into the necessary functional and familiar support. Consequently, understanding the influencing factors that affect adaptive functioning can facilitate the creation of approaches aimed at early diagnosis and intervention for ASD.

## 2. Materials and Methods

### 2.1. Procedure

The current study employed a longitudinal design. Data were retrospectively collected from an in-depth review of the files of patients who were referred to the Child and Adolescent Neuropsychiatry Unit of a third-level children’s hospital between 2019 and 2023 for a neuropsychiatric evaluation following a pediatrician’s clinical suspicion of ASD (T_0_) and for clinical follow-ups after receiving ASD diagnosis, 1 year later (T_1_). Routine assessment procedures always included neuropsychiatric examination, cognitive and adaptive functioning evaluation, assessment of ASD symptoms, and an accurate psychopathological investigation. Exclusion criteria were as follows: presence of neurological conditions (e.g., epilepsy) and presence of genetic disorders. Parents provided information about adaptive behaviors of their children and familiar demographic characteristics. Demographic data included parents’ level of education and job position. No data were collected regarding eventual children’s early intervention between the two time points. The study was conducted according to the guidelines of the Declaration of Helsinki and approved by the local Ethics Committee (protocol code: 2423_OPBG_2021, approved on 27 October 2021)

### 2.2. Participants

**Figure 1:**
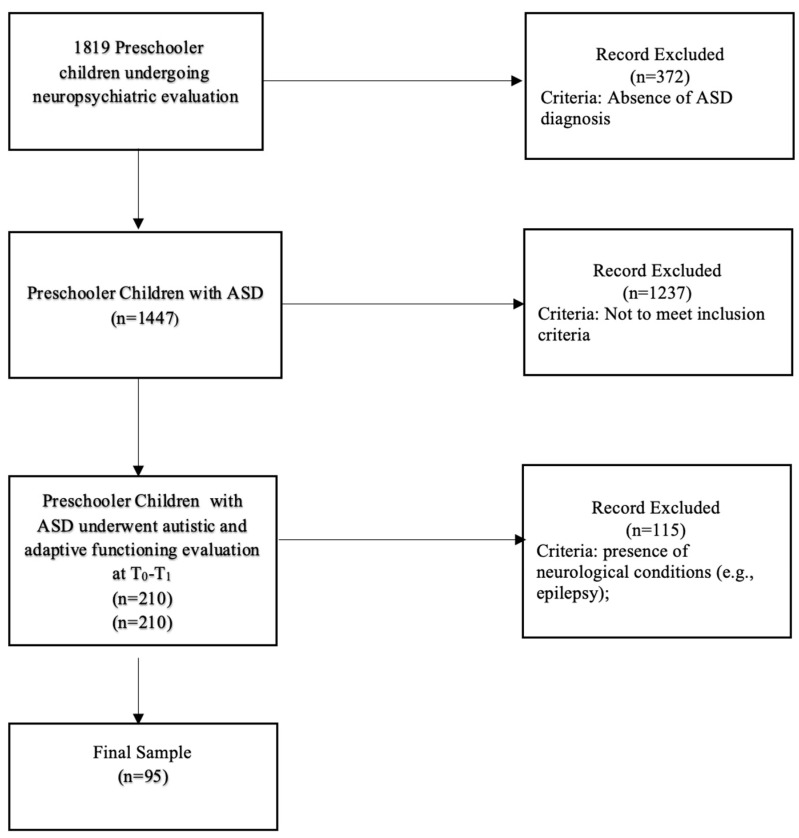
Workflow of the study.

Out of 1819 preschool-aged children undergoing neuropsychiatric evaluation, 1447 were diagnosed with ASD according to DSM-5 Criteria. After the application of the exclusion criteria, 210 preschool-aged children with ASD underwent autistic and adaptive functioning evaluation at T_0_ and T_1_. The final sample included 95 preschoolers with ASD aged between 3 and 4 years at T_0_ of both sexes (79 males and 16 females; males’ mean age at T_0_ = 3.8 SD = 1.1 and females’ mean age at T_0_ = 4.1, SD = 1.1). The sample was composed of Italian Caucasian participants. The sample reflects the epidemiological distribution of ASD in the children population in Italy [46]. Participants underwent a medical and developmental assessment, including a diagnostic evaluation performed by a multidisciplinary team composed of child psychiatrists and clinical psychologists. The workflow of the study is summarized in Figure 1.

Regarding the sociodemographic characteristics of the sample, the mother’s mean age at T_0_ was 36.8 (SD = 6.1) and the fathers’ mean age was 39.8 (SD = 6.8). To assess SES, we took into consideration the maternal and paternal levels of education and job positions. Maternal education was distributed as follows: 16.8% of mothers had a secondary school diploma, 37.9% had a high school diploma, and 41% had a university degree. Of these, 37.9% had no employment, 8.4% had a lower supervisory, technical, or similar job position, 31.6% were intermediate, small employers or own accountants, and 11.6% were a manager or professional. Concerning the fathers, 13.7% had a secondary school diploma, 54.7% had a high school diploma, and 26.3% had a university degree. In respect to the employment positions, 2.1% were unemployed, 12.6% had a lower supervisory, technical, or similar job position, 50.5% were intermediate, small employers or own accountants, and 31% were a manager or professional. 

### 2.3. Measures

#### 2.3.1. Autism Diagnostic Observation Schedule, 2nd Edition (ADOS-2 [47])

The ADOS-2 serves as a semi-structured, in-person evaluation that directly examines communication, social interaction, and the utilization of play or creativity in individuals suspected of having ASD. This assessment encompasses five distinct modules, each tailored to individuals with varying language capabilities, ranging from nonverbal to proficiently verbal. Trained clinicians were responsible for administering the ADOS-2, with the cumulative score encompassing symptoms originating from both the social affect (SA) and restricted and repetitive behaviors (RRBs) domains. The combined total calibrated severity score (TOT CSS), encompassing both the social affect calibrated severity score (SA CSS) and the RRBs CSS, was considered for the ADOS-2 assessment. ADOS-2 was performed at T_0_–T_1_. This instrument demonstrates robust interrater and test–retest reliability, along with substantial predictive capability and specificity in discriminating between ASD and non-spectrum conditions (specifically in Module 3 with a sensitivity of 0.91 and specificity of 0.84) [48,49]. Furthermore, it has been adapted for use in diverse countries. For the current study, α_SA_ = 0.83, α_RRBs_ = 0.81, and α_TOT_ = 0.81.

#### 2.3.2. Cognitive and Adaptive Functioning Measures

The cognitive or developmental level of participants was evaluated exclusively at T_0_ using the Global Developmental Quotient (GDQ) derived from the Griffiths Mental Development Scales—Extended Revised 0–2–GMDS-ER 2–8 [50]. The GMDS-ER assesses children’s developmental progress across five distinct domains (Locomotor, Personal–Social, Language, Eye and Hand Coordination, and Performance) within the age range of 0 to 8 years. Each subscale furnishes a distinct developmental quotient along with diagnostic insights into early childhood issues. The average of these quotients from the six subscales yields a Global Developmental Quotient. Children were categorized as having cognitive impairment if their GDQ was below 70 and considered without impairment if it was 70 or higher.

#### 2.3.3. Adaptive Functioning Assessment

The parents of all participants completed the Adaptive Behavior Assessment System, Second Edition (ABAS-II) to evaluate their child’s adaptive functioning [51] at both T_0_ and T_1_ time points. Depending on their child’s age, parents choose either the “0–5 years” form or the “5–21 years” form. In these forms, parents rated their child’s ability to perform various activities on a scale of 0 (indicating “not able to”) to 3 (indicating “able to do it and always performs it when needed”). This assessment covered 10 functional areas, including environment utilization, preschool skills, communication, household behavior, health and safety, play, self-care, self-control, social skills, and mobility. These areas were grouped into three main adaptive domains: conceptual (CAD), practical (PAD), and social (SAD), alongside a comprehensive score known as the General Adaptive Composite (GAC), which was calculated by summing up scaled scores from the 10 skill areas. For the analysis, composite scores from all adaptive domains (CAD, PAD, SAD, and GAC), expressed as a mean of 100 with a standard deviation of 15, were utilized. These scores were evaluated based on the ABAS-II forms used for the assessment. For the current study, α_CAD_ = 0.86, α_PADs_ = 0.82, α_SAD_ = 0.80, and α_GAC_ = 0.85.

### 2.4. Plan of Analysis

All data were uploaded to SPSS [52] version 27 for analysis. First, a Pearson correlation analysis between the keys variables was computed. Subsequently, a model was computed using multivariate analysis including bootstrap (number of samples: 1000; confidence interval level: 95.0%). The model aimed at evaluating the effects of the maternal socioeconomic status, the autistic symptomatology, the adaptive functioning, and the cognitive level at baseline (T_0_) on the adaptive functioning one year later (T_1_). Afterward, we estimated a multivariate moderation model in which the effect of all the variables at T_0_ on the adaptive functioning at T_1_ is influenced or dependent on the cognitive level.

## 3. Results

### 3.1. Clinical Indices

**Figure 2:**
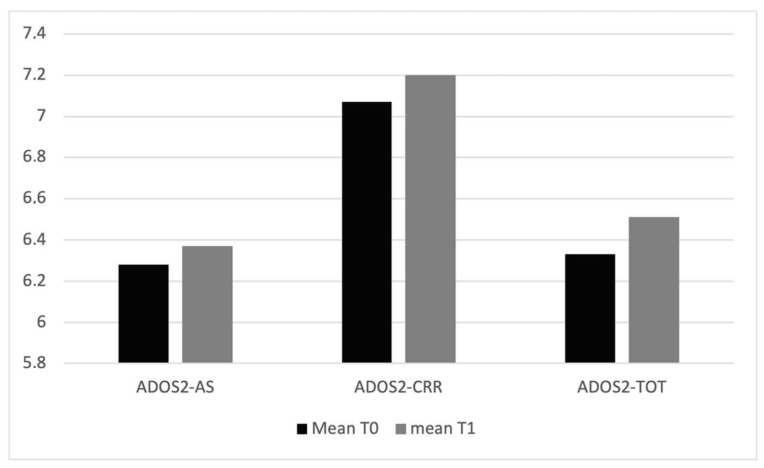
Mean values of ADOS2 scales at T_0_ and T_1_.

**Figure 3:**
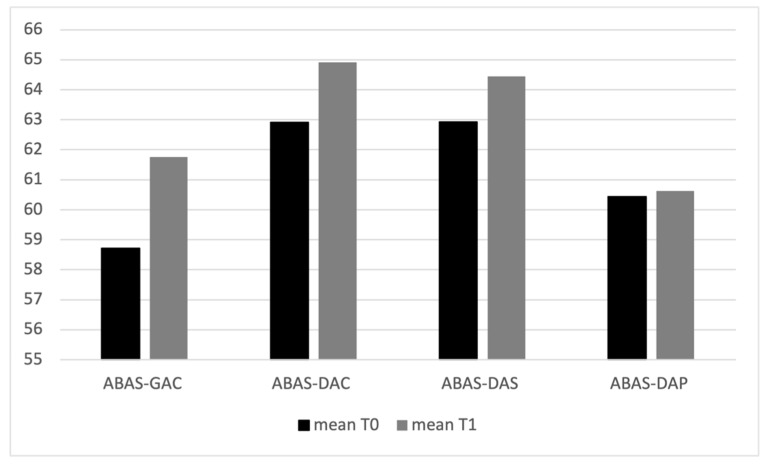
Mean values of ABAS II scales at T_0_ and T_1_.

**Figure A1:**
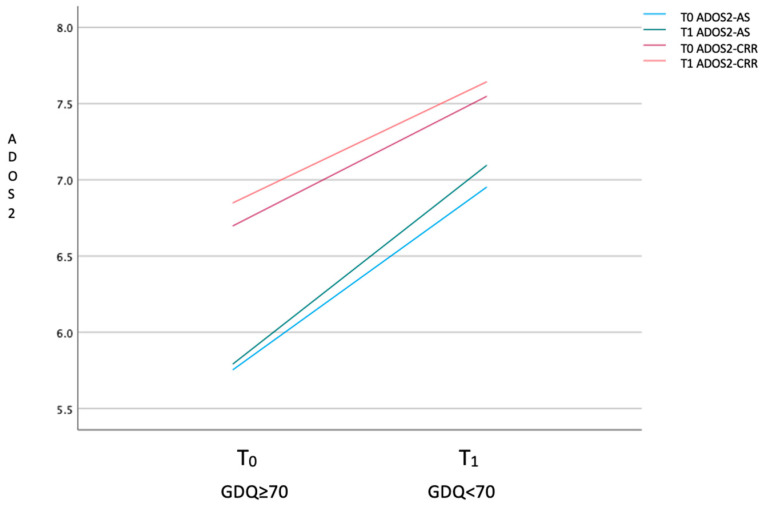
Mean of ADOS scale values of high- and low-functioning groups at T_0_ and T_1_.

**Figure A2:**
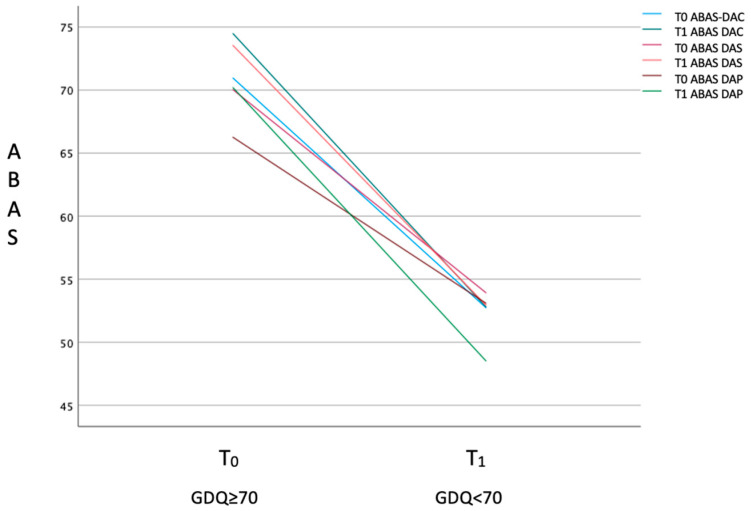
Mean of ABAS scale values of high- and low-functioning groups at T_0_ and T_1_.

Considering the variables regarding the clinical condition of the children, the mean values of ADOS-2 scale variables, AS and CRR, are higher at T_1_ than T_0_ (Table 1, Figure 2); ADOS-2 AS T_0_–T_1_ (t(94) = 42.9, *p* = 0.005); ADOS-2 T_0_–T_1_ CRR (t(94) = 51.6, *p* = 0.005); ADOS-2 TOT T_0_–T_1_ (t(94) = 46.9, *p* = 0.005). The mean values of ABAS scale variables, ABAS-GAC (t(94) = 33.7, *p* = 0.005), ABAS-DAC t(94) = 37.3, *p* = 0.005), ABAS-DAS (t(94) = 38.2, *p* = 0.005), and ABAS-DAP (t(94) = 35.6, *p* = 0.005), are higher at T_0_ than T_1_ (Table 2 and Figure 3). In particular, 19 children have, at T_0_, an ABAS-GAC higher or equal than 70 (51) (Table A1 in Appendix A). The mean of the GDQ at T_0_ is 72.18 (SD = 18.29). In particular 53 children (55.7%) have a GDQ higher or equal than 70 (M = 85.45, SD = 10.78) and 42 children (44.3%) have a GDQ lower than 70 (M = 55.43; SD = 10.23). Dividing the sample by the GDQ values higher or lower than 70, both groups show higher values of ADOS-AS than of ADOS-CRR, and both scales are higher at T_1_ (Figure A1 in Appendix A), indicating a worsening of the symptomatology. Regarding the ABAS scales, all scales are lower at T_1_, indicating a worsening of the adaptive behaviors (Figure A2 in Appendix A).

### 3.2. Correlational Analysis

To explore the relationship between the ADOS-2 scales and ABAS scales at both T_0_ and T_1_, we conducted correlational analysis. The results revealed positive correlations between all ADOS-2 scales at T_0_ and their counterparts at T_1_, as well as between all ABAS scales at T_0_ and T_1_ (Table A2 and Table A3 in Appendix A). Additionally, GDQ values exhibited positive correlations with all ABAS scales at both T_0_ and T_1_, while displaying negative correlations with ADOS-2 scales at both time points (Table A4 and Table A5 in Appendix A). Further analysis between ADOS-2 scales and ABAS scales at T_0_ revealed negative correlations between the ADOS-AS scales and ABAS scales, as well as negative correlations between ADOS-2 TOT and all ABAS scales. Notably, no significant correlations were found between ADOS CRR scales and ABAS scales at T_0_ (Table 3).

At T_1_, negative correlations were observed between all ADOS and ABAS scales (Table 4). When examining the relationship between ADOS-2 at T_0_ and ABAS at T_1_, negative correlations were found across all scales (Table 5). Similarly, analysis between ADOS-2 at T_1_ and ABAS at T_0_ revealed negative correlations between ADOS-AS and ADOS-GAC scales and all ABAS scales, while no significant correlations emerged between ADOS-CRR scales at T_1_ and ABAS scales at T_0_ (Table 6). 

A correlation emerged between the maternal education and ABAS GAC (*p* < 0.03) and ABAS DAP (*p* < 0.01) at T_1_ (Table A6 in Appendix A).

### 3.3. Multivariate Analysis

Multivariate analysis shows that ADOS-CRR T_0_ (Λ = 0.79 with F = 3.69; *p* < 0.005), ABAS-DAS T_0_ (Λ = 0.78 with F = 4.08; *p* < 0.005), ABAS-DAP T_0_ (Λ = 0.77 with F = 4.25; *p* < 0.005), and GDQ (Λ = 0.73 with F = 5.35; *p* < 0.005) have a statistically significant effect in the model. Specifically, univariate analyses highlighted that lower values of ABAS-DAS and GDQ combined with higher levels of ADOS-CRR at T_0_ were factors connected with ABAS-DAC and ABAS-DAS. In particular, higher values of ADOS-CRR at T_0_ are related to lower values of ABAS-DAC (β = −1.66, *p* < 0.05) and ABAS-DAS at T_1_ (β = −2.56, *p* < 0.1) (Table 7). Higher levels of GDQ at T_0_ have also a significant effect on all outcomes (DAC β = −0.40; *p* < 0.001; DAS β = 0.33, *p* < 0.001 DAP β = 0.34, *p* < 0.001). Moreover, no moderation effects emerged from the analysis.

## 4. Discussion

The objective of this longitudinal study was to investigate the predictive factors linked to adaptive functioning domains in preschool-aged children with ASD at two distinct time points. We considered maternal socioeconomic status, cognitive ability, and autistic symptomatology of the children as potential predictors of one-year-later specific adaptive competencies. First, considering the findings of prior research on the connection between SES and adaptive behaviors, we incorporated maternal job position and maternal education as significant variables in our study, rather than relying solely on the overall SES score [22]. In contrast with other studies [30,42,45], we found that maternal socioeconomic condition did not predict the adaptive functioning of the children. A possible explanation for this could be that, in our Italian sample, the primary responsibility for caring for a child with autism has (traditionally) rested with mothers, who are mostly unemployed and more unemployed than fathers [42], even if they are educated to a higher level [53,54]. This ambivalent condition may lead to the absence of an effect of SES on children’s adaptive functioning [42]. Nonetheless our data showed an interaction between maternal education and general adaptive functioning of the child and practical adaptive skills. The maternal educational level may influence the way in which children with ASD receive guidance for everyday life skills, and therefore have impact on adaptive skills [30]. This has a potential effect on the everyday life of the child in their home living, community use, and health and safety capacity, being a protective factor for adaptive abilities in children with ASD [42]. Regarding maternal unemployment, this condition is confirmed in the literature [55] and demonstrates the strong social impact of those syndromes and the consequent disability of the families [56], having an effect on children’s adaptive skills [57,58,59]. Focusing on the modification of the symptomatology over time, our sample showed more severe autistic symptoms in the “repetitive and restricted behavior” cluster than the “social communication and social interaction” one. Data indicated a worsening of the whole autistic symptomatology and of all adaptive functioning domains after one year. These results are in line with other longitudinal studies on preschool-aged children with ASD showing a very low percentage of improvements of autistic symptoms and adaptive functioning in the first few years after diagnosis [28,29,30,31]. As expected, low cognitive abilities, ASD symptomatology, and adaptive functioning were correlated, at both time points, showing the strong interplay between the three dimensions [5,22]. Additionally, at the first time point, the autistic symptoms in the “repetitive and restricted behavior” cluster had no relationship with the adaptive functioning of the children; instead, high symptoms in the “social communication and social interaction area” showed an inter-relationship with low adaptive competencies. After one year, both areas of autism symptomatology had a strong interaction with the adaptive functioning of the children. So, when the ASD symptomatology worsened, the adaptive functioning also worsened [60,61]. A possible explanation for the first-year results is that, at the first time point, the children had lower language and communication abilities, due to the younger age or language impairments. This condition may have had an effect on their adaptive functioning [30,31,62]. A possible explanation for the second-year results may be that RRBs appeared after the onset of the social-communicative impairment, or that, as the children aged, their RRBs may have deteriorated [63,64]. In either case, these factors would have been likely to affect the child’s measured adaptive functioning, even due to the higher environmental requests [5,65,66,67,68,69]. Finally, another possible explanation could be that, as they grew older, children who showed more problems with repetitive and restricted behavior exhibited more symptoms of internalizing and externalizing behavior, and this may have interplayed with well-being and adaptive functioning [70,71]. Regarding the possible predictors of adaptive functioning domains one year after diagnosis, we found that only repetitive and restricted behavior showed a strong relationship with social adaptive functioning and practical adaptive functioning. Focusing on the social adaptive functioning, a potential elucidation could be that the impairments or the difficulties caused by sensorial interests, mannerisms, stereotypes, or intensive and repetitive interest in specific objects at an early age influenced the engagement in the play, interactions, and social competencies of the children over time, leading to a difficult adjustment in the environment [72]. RRBs could have also impeded social learning and impacted appropriate play. Having flexibility in play is important for young children with autism to increase positive social interactions in various social settings [73]. Furthermore, it is possible that RRBs were related to social competence indirectly through emotion dysregulation [70,71]. The intersection between RRBs and emotion regulation difficulties may be important for understanding the reduced social competence associated with ASD symptomatology [74,75]. Moreover, environmental requests may have become higher during the year between measurements. In particular, the children’s entrance to kindergarten may have introduced some academic expectations, which, in turn, may have influenced the occurrence of RRBs and led to low interactions with other children [76,77]. 

Our findings also indicated a predictive role of RRBs on ABAS in the practical domain, which encompasses areas such as community use, home living, health and safety, and self-care. A possible explanation for this could be that the presence of RRBs led children to participate in significantly fewer activities specifically within the domains of community mobility and domestic chores, and set these individuals apart, resulting in an increased risk for reduced participation in everyday activities [33]. Furthermore, the domains of self-care and health and safety involve the abilities needed to carry out everyday functional tasks while ensuring their safe execution [78]. Cavkaytar and Pollard [79] showed that many individuals with ASD often require repeated instructions and exhibit challenges in autonomously performing daily life activities. An investigation delved into potential factors contributing to difficulties in the practical domain, including lack of motivation, established habits or behavioral patterns, communication limitations, sensory processing issues, and variations in performance [80]. Introducing new routines for accomplishing daily tasks can pose challenges for individuals with autism, given their tendency to engage in repetitive and stereotyped behaviors [1,33,36]. Moreover, difficulty in comprehending the task and the inability to express their own needs can impact the completion and accuracy of these tasks [80]. In the self-care domain, sensory issues related to teeth brushing or hair washing can impact the child’s practical adaptive functioning [33,36,81]. Previous studies indicated that parents of children displaying intrusive RRBs adapted their environment to accommodate their child’s needs in response to challenging behaviors associated with ASD. The research findings revealed that parents restricted their engagement in social activities and outings involving their child, thus limiting children’s community participation [82]. Is it necessary to point out that ABAS was filled in by the parents, and this may reflect the impact that RRBs have not just on the individuals displaying them, but also on caregivers, who frequently identify these behaviors as some of the most challenging to handle [83,84]. As a result, there has been a growing emphasis on interventions designed to support caregivers in the management of everyday life issues with RRBs [83,84,85]. Our findings showed that the cognitive level of a child with ASD had a strong influence on their adaptive functioning. The gap between IQ and adaptive functioning widens as individuals with autism transition from early childhood to adulthood, as indicated by research conducted by Bal et al. [26] and Tillmann et al. [5]. This observation implies that many individuals on the autism spectrum encounter challenges when it comes to translating their cognitive abilities into achieving functional independence. These data are confirmed in different longitudinal studies, underling the necessity of a proper cognitive evaluation and an early focused intervention [5,22]. Autism is a chronic condition that presents a complex medical and psychological pattern that involves the children and their family [86]. In fact, the inclusion and consideration of the heterogeneity of the autism spectrum is fundamental for research and clinical aspects [87]. Indeed, assessing the preschool-aged children with ASD symptomatology and their adaptive functioning at the time of diagnosis and after one year is relevant to understanding any possible predictors and trying to implement new approaches and help families with special monitoring during this initial, challenging period. In this regard, a parent’s account of their child’s daily functioning is crucial and helps professionals understand a child’s unique areas of strength and challenges, and helps to ensure there is consistent implementation of therapeutic techniques in the home and in community settings [88]. This last factor plays an essential role in improving the whole family’s quality of life [89].

### Limits and Future Studies

This study has potential limitations. The first is the limited sample of children with ASD, which can impact the generalization of the results to the broader population of preschool children with ASD. Second, we did not collect information regarding the intervention the children eventually underwent between the two time points. Third, our study only examined the outcomes of preschool-aged children with ASD over a relatively brief period; nonetheless, we are confident that our findings highlight a crucial period, namely, the preschool years, for the development of social orienting and behavioral issues. Moreover, our results exclusively examine the developmental effects of three specific characteristics associated with ASD and cognitive functioning on adaptive functioning. However, it is worth noting that other behavioral traits have also been linked to autistic symptomatology and adaptive functioning, such as executive functioning [90]. These and other potential confounding variables should be considered in future research. Another potential limit is the proxy parent report of the adaptive scale. Even though the ABAS II is completed autonomously by parents, it is widely used in research and clinical activities, documenting and monitoring progress over time [91]. In future studies with samples of preschoolers with ASD, it would be advisable to compare a test that parents fill out on their own with a parental interview. Despite these limitations, this study has important strengths, such as the heterogeneity and representativeness of the samples, in which we included all levels of cognitive functioning, the male–female ratio, and the collection of the family conditions in the Italian population [38]. The use of this information can provide care and alleviate some of the challenges experienced by the children with ASD, their parents, and the health care providers, most of all in the preschool period or after the diagnosis. Future research should thoroughly study the family’s protective and risk factors for adaptive functioning in different geographic areas to provide more elements for the recovery and support of this population. Furthermore, future studies should aim to explore the impact of early social interest and behavioral problems across an extended developmental timeframe to gain a comprehensive understanding of their implications for later development and better differentiate the adaptive difficulties and necessities of this specific stage of life. 

## Figures and Tables

**Table 1:** Mean values of ADOS scales at T_0_ and T_1_.

	*Minimum*	*Maximum*	*Mean*	*SD*
T_0_ ADOS2-AS	3	10	6.28	1.596
T_0_ ADOS2-CRR	4	10	7.07	1.511
T_0_ ADOS2-TOT	4	9	6.33	1.267
T_1_ ADOS2-AS	3	11	6.37	1.444
T_1_ ADOS2-CRR	4	10	7.20	1.357
T_1_ ADOS2-TOT	4	9	6.51	1.352

**Table 2:** Mean values of ABAS scales at T_0_ and T_1_.

	*Minimum*	*Maximum*	*Mean*	*SD*
T_0_ ABAS-GAC	37	97	58.71	12.672
T_0_ ABAS-DAC	43	115	62.91	15.183
T_0_ ABAS-DAS	45	121	62.92	14.482
T_0_ ABAS-DAP	41	96	60.44	11.969
T_1_ ABAS-GAC	37	111	61.75	17.852
T_1_ ABAS-DAC	41	111	64.91	16.919
T_1_ ABAS-DAS	40	116	64.44	16.408
T_1_ ABAS-DAP	40	106	60.62	16.586

**Table 3:** Pearson correlation coefficient for selected variables from ADOS2 scales and ABAS scales at T_0_.

	T_0_ ABAS-GAC	T_0_ ABAS-DAC	T_0_ ABAS-DAS	T_0_ ABAS-DAP
T_0_ ADOS2-AS	−0.288 **	−0.287 **	−0.228 *	−0.224 *
T_0_ ADOS2-CRR	−0.177	−0.099	−0.200	−0.161
T_0_ ADOS2-TOT	−0.388 **	−0.359 **	−0.310 **	−0.342 **

* *p* < 0.05; ** *p* < 0.01.

**Table 4:** Pearson correlation coefficient for selected variables ADOS2 scales at T_1_ and ABAS scales at T_1_.

	T_1_ ABAS-GAC	T_1_ ABAS-DAC	T_1_ ABAS-DAS	T_1_ ABAS-DAP
T_1_ ADOS2-AS	−0.337 **	−0.402 **	−0.305 **	−0.263 *
T_1_ ADOS2-CRR	−0.285 **	−0.293 **	−0.283 **	−0.383 **
T_1_ ADOS2-TOT	−0.400 **	−0.474 **	−0.333 **	−0.388 **

* *p* < 0.05; ** *p* < 0.01.

**Table 5:** Pearson correlation coefficient for selected variables from ADOS2 scales at T_0_ and ABAS scales at T_1_.

	T_1_ ABAS-GAC	T_1_ ABAS-DAC	T_1_ ABAS-DAS	T_1_ ABAS-DAP
T_0_ ADOS2-AS	−0.337 **	−0.336 **	−0.288 **	−0.325 **
T_0_ ADOS2-PC	−0.258 *	−0.271 **	−0.391 **	−0.275 **
T_0_ ADOS2-TOT	−0.431 **	−0.433 **	−0.399 **	−0.419 **

* *p* < 0.05; ** *p* < 0.01.

**Table 6:** Pearson correlation coefficient for selected variables from ABAS scales at T_0_ and ADOS2 scales at T_1_.

	T_1_ ADOS2-AS	T_1_ ADOS2-CRR	T_1_ ADOS2-TOT
T_0_ ABAS-GAC	−0.296 **	−0.185	−0.362 **
T_0_ ABAS-DAC	−0.273 **	−0.125	−0.331 **
T_0_ ABAS-DAS	−0.280 **	−0.091	−0.297 **
T_0_ ABAS-DAP	−0.261 *	−0.212 *	−0.320 **

* *p* < 0.05; ** *p* < 0.01.

**Table 7:** Summary of the model predicting ABAS scales at T_1_ toward ABAS scales and ADOS2 scales at T_0_.

	T_1_ ABAS DAC		T_1_ ABAS DAS		T_1_ ABAS DAP	
	*F*	*B (SE)*	*F*	*B (SE)*	*F*	*B (SE)*
Corrected model	22.85	/	13.46	/	15.61	/
Intercept	1.02	12.53 (12.37)	4.46	29.42 (13.92)	0.91	12.88 (13.45)
Maternal job	5.43	2.45 (1.05)	3.73	2.28 (1.18)	3.45	2.12 (1.14)
Maternal scholarity	1.32	1.23 (1.58)	0.862	−0.45 (2.24)	0.30	0.64 (2.24)
T_0_ ADOS2 AS	0.01	−0.10 (0.82)	0.11	0.31 (0.92)	0.49	−0.62 (0.89)
T_0_ ADOS2 CRR	4.41	−1.66 * (0.79)	8.27	−2.56 ** (0.89)	3.21	−1.5 (0.86)
T_0_ ABAS DAC	3.00	0.22 (0.12)	0.01	0.015 (0.14)	0.00	−0.01 (0.14)
T_0_ ABAS DAS	5.61	0.34 * (0.14)	9.70	0.51 ** (0.16)	0.30	0.08 (0.15)
T_0_ ABAS DAP	0.08	−0.05 (0.18)	0.50	−0.14 (0.20)	6.54	0.51 ** (0.20)
GDQ	20.96	0.40 *** (0.08)	11.35	0.33 *** (0.09)	13.03	0.34 *** (0.09)
R^2^ (adjusted)	0.67		0.55		0.58	

* *p* < 0.05; ** *p* < 0.01; *** *p* < 0.001.

## Data Availability

The data that support this funding are not available for ethical restrictions.

## Appendix A

**Table A1:** ABAS-GAC higher than 70. Descriptive statistics of ABAS scales at T_0_ and T_1_.

	*Minimum*	*Maximum*	*Mean*	*SD*
T_0_ ABAS-GAC	70	97	78.16	7.904
T_0_ ABAS-DAC	59	115	84.11	13.424
T_0_ ABAS-DAS	68	121	83.89	13.552
T_0_ ABAS-DAP	66	96	76.16	8.288
T_1_ ABAS-GAC	52	111	80.26	17.508
T_1_ ABAS-DAC	59	111	81.74	16.312
T_1_ ABAS-DAS	45	116	77.74	19.419
T_1_ ABAS-DAP	50	106	74.79	14.551

**Table A2:** Pearson correlation coefficient for selected variables from ADOS2 scales at T_0_ and ADOS2 scales at T_1_.

	**T_1_ ADOS2 AS**	**T_1_ ADOS2 CRR**	**T_1_ ADOS2 TOT**
T_0_ ADOS2 AS	0.379 **	0.381 **	0.475 **
T_0_ ADOS2 CRR	0.382 **	0.325 **	0.378 **
T_0_ ADOS2 TOT	0.532 **	0.518 **	0.617 **

** *p* < 0.01.

**Table A3:** Pearson correlation coefficient for selected variables from ABAS scales at T_0_ and T_1_.

	T_1_ ABAS-GAC	T_1_ ABAS-DAC	T_1_ ABAS-DAS	T_1_ ABAS-DAP
T_0_ ABAS-GAC	0.706 **	0.680 **	0.558 **	0.657 **
T_0_ ABAS-DAC	0.698 **	0.691 **	0.510 **	0.610 **
T_0_ ABAS-DAS	0.687 **	0.673 **	0.606 **	0.616 **
T_0_ ABAS-DAP	0.653 **	0.609 **	0.501 **	0.660 **

** *p* < 0.01.

**Table A4:** Pearson correlation coefficient for GDQ and selected variables from ABAS at T_0_.

ABAS	GDQ
T_0_ ABAS-GAC	0.629 **
T_0_ ABAS-DAC	0.635 **
T_0_ ABAS-DAS	0.533 **
T_0_ ABAS-DAP	0.526 **
T_1_ ABAS-GAC	0.672 **
T_1_ ABAS-DAC	0.688 **
T_1_ ABAS-DAS	0.595 **
T_1_ ABAS-DAP	0.622 **

** *p* < 0.01.

**Table A5:** Pearson correlation coefficient for GDQ and selected variables from ADOS-2 scales at T_0_ and T_1_.

	GDQ
T_0_ ADOS2-AS	−0.418 **
T_0_ ADOS2-CRR	−0.229 *
T_0_ ADOS2-TOT	−0.517 **
T_1_ ADOS2-AS	−0.443 **
T_1_ ADOS2-CRR	−0.311 **
T_1_ ADOS2-TOT	−0.490 **

* *p* < 0.05; ** *p* < 0.01.

**Table A6:** Pearson correlation coefficient for selected variables maternal scholarity and job and ABAS at T_1_.

	T_1_ ABAS-GAC	T_1_ ABAS-DAC	T_1_ ABAS-DAS	T_1_ ABAS-DAP
Maternal scholarity	0.034 *	0.090	−0.008	0.019 *
Maternal job	0.163	0.174	0.160	0.154

* *p* < 0.05.
